# Black soldier fly and gut health in broiler chickens: insights into the relationship between cecal microbiota and intestinal mucin composition

**DOI:** 10.1186/s40104-019-0413-y

**Published:** 2020-02-03

**Authors:** Ilaria Biasato, Ilario Ferrocino, Sihem Dabbou, Rocchina Evangelista, Francesco Gai, Laura Gasco, Luca Cocolin, Maria Teresa Capucchio, Achille Schiavone

**Affiliations:** 1 Department of Agricultural, Forest and Food Sciences, University of Turin, Largo Paolo Braccini 2, 10095 Grugliasco, TO Italy; 2 Department of Veterinary Sciences, University of Turin, Largo Paolo Braccini 2, 10095 Grugliasco, TO Italy; 3 Institute of Science of Food Production, National Research Council, Largo Paolo Braccini 2, 10095 Grugliasco, TO Italy

**Keywords:** 16S rRNA, Gut health, *Hermetia illucens* L., Histochemistry, Insect meal, Microbiota, Mucin, Poultry

## Abstract

**Background:**

The relationship between diet and intestinal microbiota and mucin composition appears to be fundamental for poultry gut health. The effects of insect meal (whose role as alternative feed ingredient is now well recognized) on gut microbiota and mucin composition have recently been reported in *Tenebrio molitor*-fed free-range and broiler chickens, but no data are currently available for *Hermetia illucens* (HI)-fed broilers. The present study evaluated the effects of dietary HI meal inclusion on cecal microbiota and intestinal mucin composition of broiler chickens.

**Results:**

A total of 256 male broiler chickens were allotted to 4 dietary treatments (control diet [C] and 5%, 10% and 15% HI meal inclusion, with 8 replicate pens/treatment and 8 birds/pen) and slaughtered at 35 d of age (2 animals/pen, 16 birds/diet). The cecal microbiota assessment by 16S rRNA amplicon based sequencing showed lower alpha diversity in HI15 chickens (Shannon, *P* < 0.05) and higher beta diversity (Adonis and ANOSIM, *P* < 0.001) in birds fed HI diets than C. Furthermore, HI15 birds displayed significant increase of the relative abundance of Proteobacteria phylum (False Discovery Rate [FDR] <  0.05) when compared to HI10. L-*Ruminococcus* (*Ruminococcus* from Lachnospiraceae family), *Faecalibacterium*, *Blautia* and *Clostridium* genera were found to be characteristic of HI5 cecal microbiota (FDR < 0.05), while broiler chickens fed HI10 and HI15 diets were characterized (FDR < 0.05) by *Lactobacillus* and *Ruminococcus* (HI10) and *Bacteroides*, *Roseburia* and *Helicobacter* genera (HI15). Periodic-acid Schiff, Alcian Blue pH 2.5 and high iron diamine staining on small and large intestine also demonstrated lower mucin staining intensity in the intestinal villi of HI10 and HI15 birds than C (*P* < 0.05).

**Conclusions:**

Dietary HI meal utilization at low inclusion levels (i.e., 5%) positively influenced either the cecal microbiota or the gut mucin dynamics in terms of selection of potentially beneficial bacteria and increase in villi mucins. However, high inclusion levels (in particular the 15%) may have a negative influence in terms of partial reduction of microbial complexity, reduction of potentially beneficial bacteria, selection of bacteria with mucolytic activity and decrease in villi mucins.

## Background

Optimal gastrointestinal health and functionality is essential for sustainable animal production, since it has direct repercussions on both the animal health and the performance [1]. The gut barrier (comprising the microbiota and their products, mucus layers, host-derived antimicrobial compounds, epithelium, and underlying immune tissue) constantly interacts with the dietary nutrients, in order to maintain the delicate balance needed for preventing the passage of harmful microorganisms and substances into the animal body [2]. In particular, the relationship between the diet and the gut microbiota and mucin dynamics appears to be fundamental. As a first aspect to consider, one of the main defense components of the gastrointestinal environment against the enteric pathogens is represented by the intestinal microbiota. Indeed, imbalances of gut microbiota–host interaction are frequently associated with several intestinal disorders [3]. In parallel, the mucus layers represent the first host-derived line of defense in the intestine [4]. Mucus, which is mainly composed of mucins, traps the pathogenic bacteria and promotes their expulsion from the intestine via the luminal flow, as well as having a lubricant activity, modulates the digestion and absorption of the nutrients, and provides the colonization sites and nutrients for the commensal microbes [5, 6]. Bacterial colonization and proliferation has been reported to have a key role in determining the mucin composition, both by the synthesis of mucin-specific glycosidases, glycosulfatases and proteases [7, 8] and the modulation of mucin gene expression [9]. There is also evidence that several feed substances may widely affect the complex, delicate relationship existing between the gut microbiota and mucin dynamics in poultry, either by directly modifying the intestinal mucin composition [10, 11], or indirectly by modulating the intestinal microbial population [12, 13].

Insects as novel, alternative feed ingredients has now become a worldwide, well-recognized research topic in animal nutrition, because of their excellent nutritive properties and peculiar rearing characteristics [14, 15]. Indeed, insects contain high quality and quantity of protein [14] and they can easily be reared on several organic side streams, thus reducing their environmental and economic impact and allowing their transformation into high-protein feeds [15]. In particular, yellow mealworm (*Tenebrio molitor*, TM) and black soldier fly (*Hermetia illucens*, HI) larvae are characterized by a remarkable nutritional profile in terms of crude protein (CP: 52.8 ± 4.2% [TM] and 42.1 ± 1.0% [HI]) and ether extract (EE: 36.1 ± 4.1 [TM] and 26.0 ± 8.3 [HI]) contents that make them extremely promising for poultry feeding [14]. Dietary TM meal inclusion has recently been reported to significantly influence the gut health of free-range [16] and broiler [17] chickens, in particular by affecting both their cecal microbiota (in terms of modified phylum and genus profile) and their intestinal mucin dynamics (in terms of altered villi mucins). However, no data about the modulation of gut microbiota and mucin composition by HI meal utilization are currently available in poultry.

Based on these considerations, the present study investigates the effects of dietary HI larva meal inclusion on cecal microbiota and intestinal mucin composition of broiler chickens.

## Methods

### Birds and experimental design

The experimental design of the present study is described in details in the research published by Dabbou et al. [18]. Briefly, a total of 256 1-day-old male broiler chicks (Ross 708) were randomly distributed into four isonitrogenous and isoenergetic dietary treatments, each consisting of 8 pens as replicates (1.0 m wide × 1.5 m long, equipped with a feeder, an automatic drinker and rice hulls as bedding) with 8 chicks per pen. The control diet (C) was based on maize meal, corn gluten meal and soybean meal, while the experimental diets were obtained including 5%, 10% and 15% of a partially defatted HI larva meal (Hermetia Deutschland GmbH & Co. KG, Baruth/Mark, Germany) as partial replacement of soybean meal, corn gluten meal and soybean oil (HI5, HI10 and HI15 groups, respectively). The chemical composition of the HI meal was as follows: 942 g/kg of diet of dry matter (DM), 553 g/kg DM of crude protein (CP), 180 g/kg DM of ether extract (EE) and 24.4 MJ/kg DM of gross energy. Detailed information about the diets is summarized in Additional file 1. Nutrients digestibility, apparent metabolizable energy (AME) and apparent metabolizable energy corrected for nitrogen balance (AMEn) of the HI meal used for feed formulation were previously assessed [19]. All the birds were reared under the same environmental conditions (lighting schedule: 18 h light: 6 h dark; T: 32 °C during the first day, with reduction by 4 °C per week according to the age of the broilers until it reached 20 °C) throughout the whole experimental trial and fed ad libitum. The chickens received regular vaccination against Newcastle disease, Marek disease, infectious bronchitis and coccidiosis at hatching. The growth performance of the broiler chickens were also evaluated throughout the experimental trial, as reported in details by Dabbou et al. [18]. Briefly, the live weight (LW), the average daily gain (ADG), the average daily feed intake (DFI) and the feed conversion ratio (FCR) of the birds increased with increasing levels of dietary HI meal inclusion (LW: end of the starter, the grower and the finisher periods; ADG: starter and grower periods; DFI: starter period; FCR: grower and finisher periods, and overall). The experimental period lasted 35 d.

### Intestinal sampling and processing

A total of 16 chickens per treatment (2 birds/pen) were randomly selected and slaughtered in a commercial abattoir at the end of the experimental trial. Cecal content was sampled into sterile plastic tubes with a sterile spatula, immediately refrigerated (for a maximum of 2 h) and frozen at − 80 °C until DNA extraction. Well-defined, standardized segments of both the small (duodenum, jejunum and ileum) and the large (cecum) intestine were sampled and processed for histochemical staining, as previously reported by Biasato et al. [16].

### DNA extraction and sequencing

A pool of the cecal content from 2 chickens per pen (8 pools per feeding group) was submitted to DNA extraction and sequencing. The DNA was extracted with the RNeasy Power Microbiome KIT (Qiagen, Milan, Italy) following the instructions reported by the manufacturer. One μL of RNase (Illumina Inc., San Diego, CA) was added to digest the RNA in the DNA samples, with an incubation of 1 h at 37 °C. The DNA was quantified using the NanoDrop and standardized at 5 ng/μL. Due to poor DNA quality, one samples belonging to the HI5 group was excluded. The extracted DNA was used to assess the microbiota by the amplification of the V3-V4 region of the 16S rRNA gene using the following primers: 16S-F (5′- TCG TCG GCA GCG TCA GAT GTG TAT AAG AGA CAG CCT ACG GGN GGC WGC AG-3′) and 16S-R (5′-GTC TCG TGG GCT CGG AGA TGT GTA TAA GAG ACA GGA CTA CHV GGG TAT CTA ATC C-3′) [20]. Twenty-five μL PCR reactions were prepared using 12.5 μL of the 2X KAPA HiFi HotStart Ready Mix Taq (Kapa Biosystems, Wilmington, MA), 1 μmol/L of each primer and 2.5 μL of DNA. A total of 25 cycles of 30 s of denaturation (95 °C), 30 s of primer annealing (55 °C) and 30 s of primer elongation (72 °C), followed by a final elongation step (72 °C) of 5 min, were carried out. The PCR products were purified by means of an Agencourt AMPure kit (Beckman Coulter, Milan, Italy) and the resulting products were tagged by using the Nextera XT Index Kit (Illumina Inc., San Diego, CA) according to the guidelines reported by the manufacturer. Sequencing was performed with a MiSeq Illumina instrument (Illumina) with V3 chemistry and generated 250 bp paired-end reads according to the manufacturer’s instructions. The software used for the base-calling and Illumina barcode demultiplexing processes were the MiSeq Control Soft. V2.3.0.3, the RTA v1.18.42.0 and the CASAVA v1.8.2.

### Histochemical staining

The intestinal sections of 16 chickens per dietary treatment (2 birds/pen) were submitted to three different histochemical staining, as previously reported by Biasato et al. [16]: periodic-acid Schiff (for the identification of the neutral mucins), Alcian Blue pH 2.5 (for the identification of the acidic sialylated mucins) and high iron diamine (for the identification of the acidic sulfated mucins).

### Mucin staining intensity

The mucin staining intensity of the goblet cells was assessed on one slide per histochemical staining for each intestinal segment by using a well-defined, semiquantitative score, according to Biasato et al. [16].

### Bioinformatics and statistical analysis

Paired-end reads were merged by FLASH software [21] with default parameters. QIIME 1.9.0 was used for quality filtered (at Phred < Q20) [22] and the recently described pipeline [23] was adopted. The Operational Taxonomic Units (OTUs) clustering was performed at 97% of similarity [24] and centroids sequence were used to assign taxonomy by the Greengenes 16S rRNA gene database (version 2013). Alpha diversity indices were calculated using the *diversity* function of the vegan package [25]. Diet-related differences were assessed by pairwise t-test, Kruskal-Wallis tests or Wilcoxon rank sum test as appropriate. *P* values were adjusted for multiple testing and a false discovery rate (FDR) < 0.05 was considered statistically significant. Weighted UniFrac distance matrices were used to perform Adonis and ANOSIM statistical tests in the R environment (https://www.r-project.org). A filtered OTU table was generated at 0.1% abundance in at least 2 samples through QIIME. The so-obtained table was used to determine the Principal component analysis (PCA) in the R environment. The OTU table displayed the highest taxonomy resolution reached by the 16S data. Indeed, when the genus level was not reached by the taxonomy assignment, the bacterial family, order or phyla were actually showed.

The statistical analysis of the histochemical data was performed using IBM SPSS Statistics V20.0.0 software. The histochemical scores were analyzed using the generalized linear model (GLM) recently adopted by Biasato et al. [16]. Results were expressed as least squares means and SEM. *P* values < 0.05 were considered statistically significant.

## Results

### Cecal microbiota characterization

A total of 1,716,304 raw reads (2 × 250 bp) were obtained after sequencing. After joint and quality filtering, a total of 1,602,517 reads passed the filters applied through QIIME, with an average value of 69,674 reads/sample (SD: 21,342) and a median sequence length of 441 bp. In order to avoid potential biases due to different sequencing depths, all the samples were rarefied at 3600 reads after the raw read quality filtering. The rarefaction analysis and the Good’s coverage indicated a satisfactory coverage for all the samples (average Good’s coverage of 94%).

**Fig. 1:**
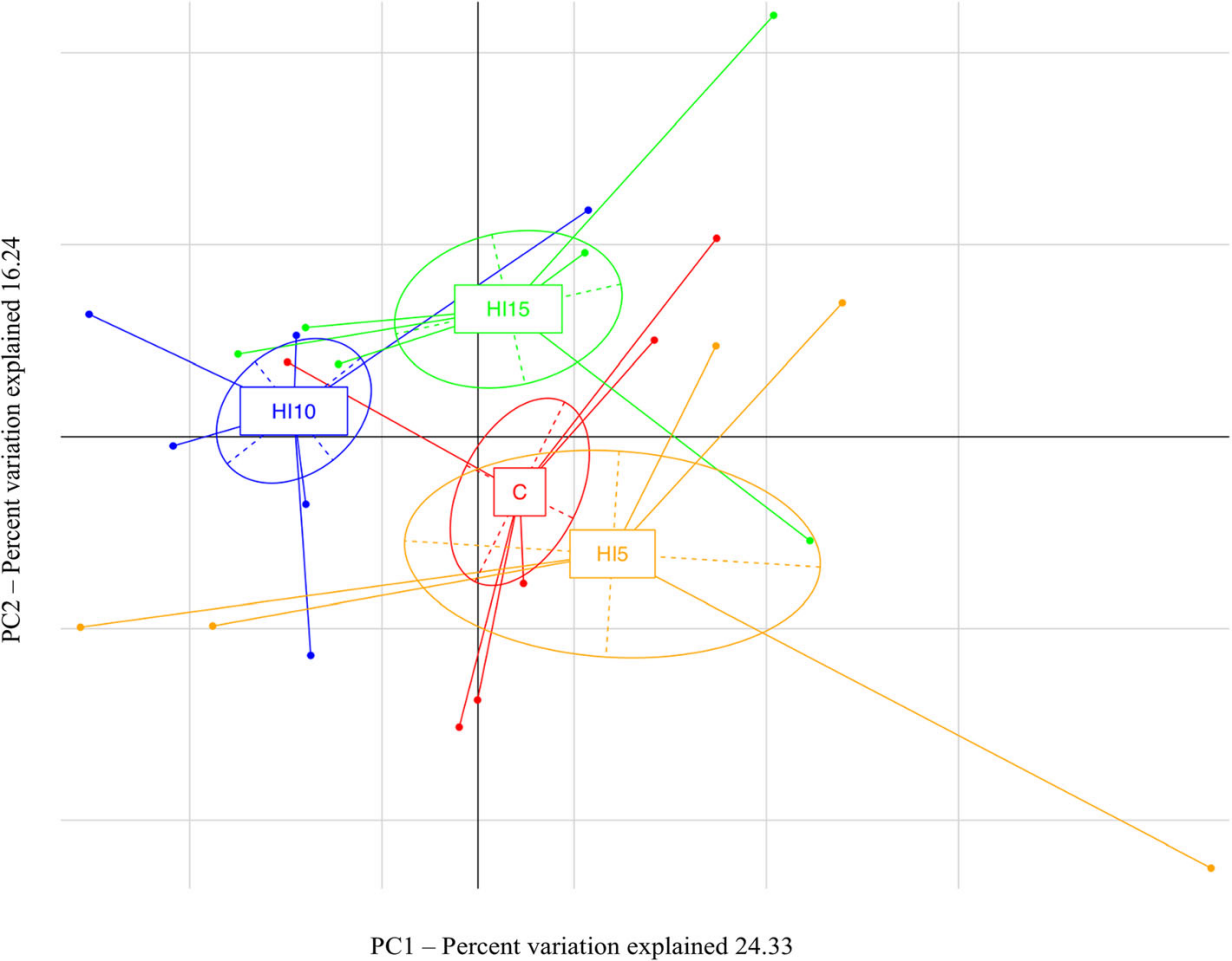
Bacterial community composition (weighted UniFrac beta diversity, PCA plots) in cecal samples of broiler chickens fed control (C), 5% (HI5), 10% (HI10) and 15% (HI15) inclusion level of *Hermetia illucens* meal diets

Dietary HI meal inclusion significantly affected the diversity within the microbial populations, as indicated by a lower Shannon index (*P <* 0.05) observed in the broiler chickens fed HI15 (6.49) compared to the other diets (C = 7.25; HI5 = 6.88; HI10 = 7.36). However, the other alpha diversity measures showed no significant differences (*P* > 0.05) among the C (average PD Whole Tree: 79.29; average Chao1: 2680.28; average observed species richness: 1161.33), HI5 (average PD Whole Tree: 73.93; average Chao1: 2418.11; average observed species richness: 1024.80), HI10 (average PD Whole Tree: 77.42; average Chao1: 2318.36; average observed species richness: 1085.50) and HI15 groups (average PD Whole Tree: 69.23; average Chao1: 2160.36; average observed species richness: 956.33). Adonis and ANOSIM statistical tests based on Weighted UniFrac distance matrix showed significant differences between the C and the HI groups in the relative abundance of the microbial species (*P* <  0.001). Indeed, the PCA showed a clear shift of the cecal microbiota as a function of the dietary HI meal inclusion (Fig. 1).

**Fig. 2:**
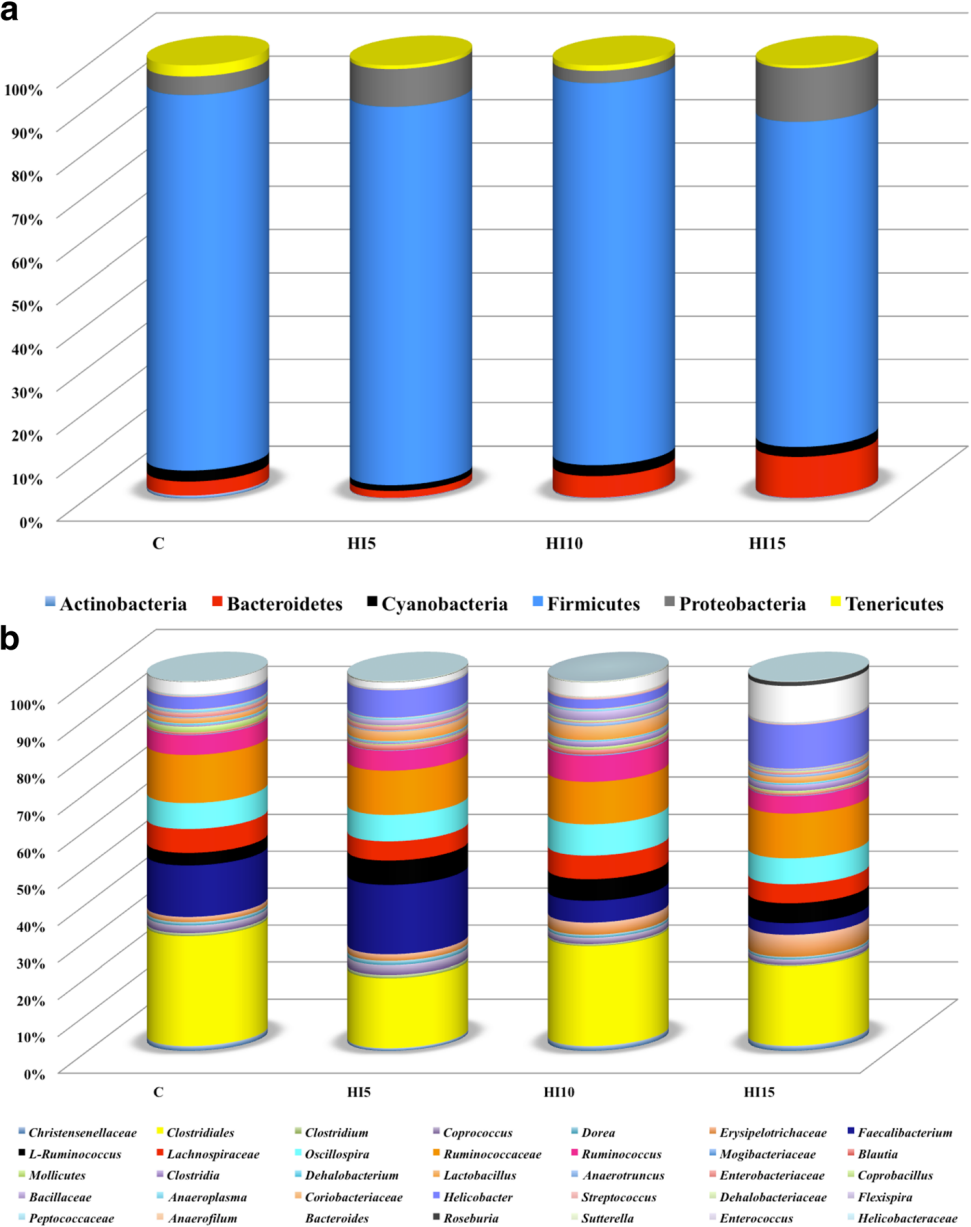
Relative abundance of the main bacterial phyla (**a**) and genera (**b**) in cecal samples of broiler chickens fed control (C), 5% (HI5), 10% (HI10) and 15% (HI15) inclusion level of *Hermetia illucens* meal diets

Relative abundances of the main phyla and genera in the broiler chickens of the present study obtained by 16S rRNA gene sequencing are summarized in Fig. 2 and Additional file 2. Firmicutes represented the dominant phylum of the cecal microbiota in either the C or the HI groups, outnumbering the Proteobacteria and Bacteoidetes phyla (Fig. 2a, Additional file 2). Within the phylum Firmicutes, Unclassified members (U. m.) of Clostridiales order, U. m. of Ruminococcaceae family, *Faecalibacterium, Oscillospira*, U. m. of Lachnospiraceae family, *Ruminococcus*, L-*Ruminococcus* (*Ruminococcus* belonging to Lachnospiraceae family), U. m. of Erysipelotrichaceae family and *Lactobacillus* were identified as the main OTUs in the birds fed both the C and the HI diets (Fig. 2b). *Helicobacter* was the dominant member of the Proteobacteria phylum in either the C or the HI groups (Fig. 2b). Within the phylum Bacteroidetes, *Bacteroides* was observed as predominant OTU in the animals fed both the C and the HI diets (Fig. 2b, Additional file 2).

**Fig. 3:**
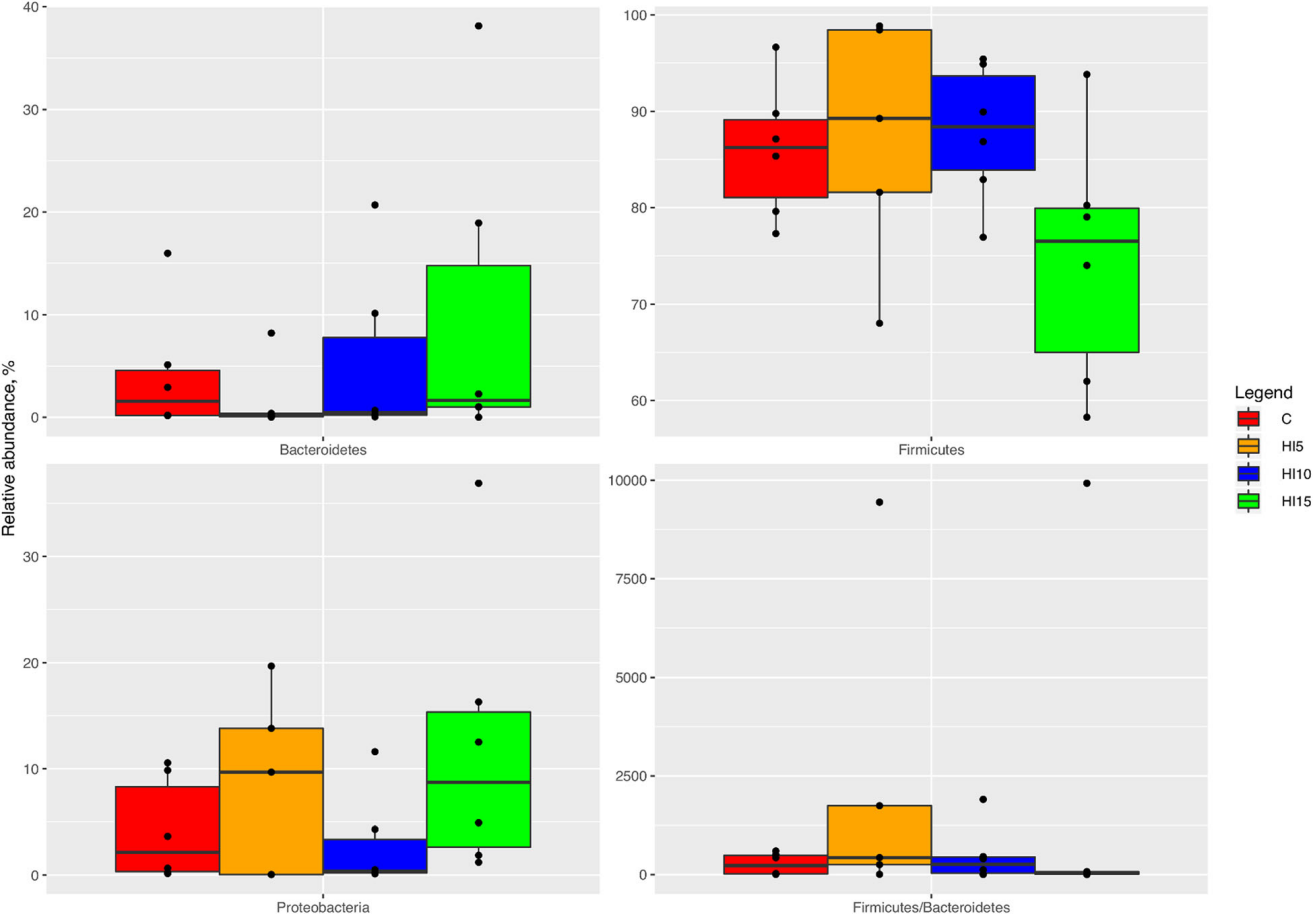
Relative abundance at phylum level of differentially abundant OTUs in cecal samples of broiler chickens fed control (C), 5% (HI5), 10% (HI10) and 15% (HI15) inclusion level of *Hermetia illucens* meal diets. Pairwise Kruskal-Wallis test, FDR < 0.05

Compared to the C group (Fig. 3), the birds fed HI displayed unaffected relative abundances of Firmicutes and Bacteroidetes phyla (FDR *>* 0.05). On the contrary, the relative abundance of Proteobacteria was higher in the HI15 animals than the HI10 (FDR < 0.05). The birds fed HI also showed unaffected Firmicutes:Bacteroidetes ratios compared to the C group (FDR < 0.05). Comparing the relative abundances of the main OTUs among the dietary treatments, a specific microbiota signature was observed for each diet. In particular, the broiler chickens fed C were characterized by the presence of U. m. of Lachnospiraceae family (FDR < 0.05), while L-*Ruminococcus* (*Ruminococcus* from Lachnospiraceae family), *Faecalibacterium*, *Blautia* and *Clostridium* genera were found to be characteristic of the HI5 diet (FDR < 0.05). The broiler chickens fed HI10 were characterized (FDR < 0.05) by *Lactobacillus* and *Ruminococcus* OTUs, whereas *Bacteroides*, *Roseburia* and *Helicobacter* genera were characteristic for the HI15 diet (FDR < 0.05).

### Intestinal mucin composition

**Fig. 4:**
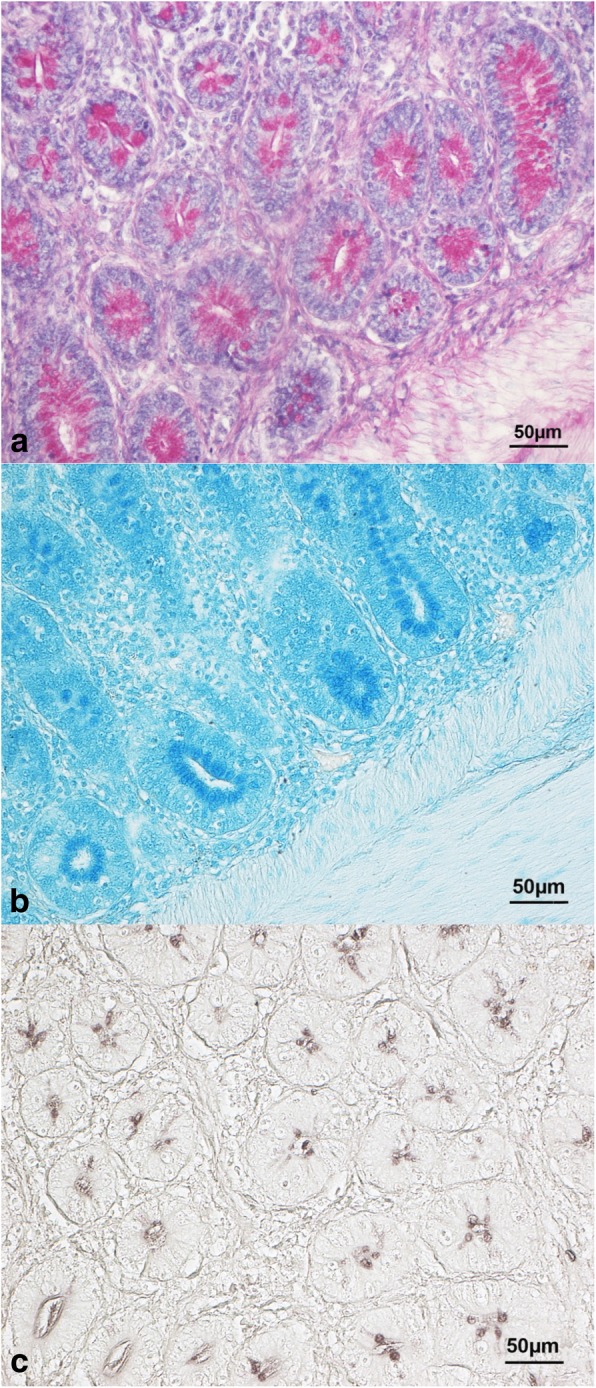
Histological pictures of (**a**) duodenal crypts stained with periodic-acid Schiff (C group, 40× magnification), (**b**) jejunal crypts stained with Alcian Blue pH 2.5 (HI5 group, 40× magnification) and (**c**) ileal crypts stained with high iron diamine (HI10 group, 40× magnification). Crypts show higher neutral and acidic sialylated mucin staining intensity than the acidic sulfated

The mucin type (*P* < 0.001), the gut segment (*P* < 0.001) and the crypt fragment (*P* < 0.001) significantly influenced the mucin staining intensity in the intestinal crypts, while the histochemical findings were unaffected by dietary HI meal inclusion (*P* > 0.05, Table 1). In particular, the crypts showed higher neutral and acidic sialylated mucins staining intensity (*P* < 0.001) than the acidic sulfated (Fig. 4). Higher mucin staining intensity was also found in the ileal crypts (*P* < 0.001) when compared to the other gut segments. Furthermore, the crypt base showed greater mucin staining intensity (*P* < 0.001) than the midsection and tip, with a significant decrease (*P* < 0.001) being also observed from the midsection to the tip (Table 2).

**Table 1:** Effects of diet, mucin type, gut segment and crypt-villus fragment on mucin staining intensity in the broiler chickens

Factor	d.f.^f^	Chi-square	P^*^
Crypts			
Diet^a^	3	3.751	0.290
Mucin type^b^	2	22.566	< 0.001
Gut segment^c^	3	63.140	< 0.001
Fragment^d^	2	247.461	< 0.001
Villi			
Diet	3	25.497	< 0.001
Mucin type	2	4.510	0.100
Gut segment^e^	2	571.512	< 0.001
Fragment	2	1.488	0.475

^a^Four dietary treatments: C = control; HI5 = 5% inclusion level of *Hermetia illucens*; HI10 = 10% inclusion level of *Hermetia illucens*; HI15 = 15% inclusion level of *Hermetia illucens*

^b^Three types: neutral, acidic sialylated and acidic sulfated mucins

^c^Four gut segments: duodenum, jejunum, ileum and caecum

^d^Three fragments: base, midsection and tip

^e^Three gut segments: duodenum, jejunum and ileum

^f^Degrees of freedom

^*^Statistical significance: *P* < 0.05. Statistical trend: *P* ≤ 0.10

**Table 2:** Least square means of mucin staining intensity in the intestinal crypts of the broiler chickens in relation to diet, mucin type, gut segment and crypt fragment

	Factor	Factor levels	Mucin staining intensity^1,2^
Crypts	Diet	C	1.23 ± 0.03
		HI5	1.27 ± 0.03
		HI10	1.27 ± 0.03
		HI15	1.30 ± 0.03
	Mucin type	Neutral	1.32 ± 0.03^A^
		Acidic sialylated	1.31 ± 0.02^A^
		Acidic sulfated	1.18 ± 0.02^B^
	Gut segment	Duodenum	1.26 ± 0.03^B^
		Jejunum	1.20 ± 0.03^BC^
		Ileum	1.45 ± 0.03^A^
		Caecum	1.19 ± 0.03^C^
	Fragment	Base	1.59 ± 0.03^A^
		Midsection	1.17 ± 0.02^B^
		Tip	1.10 ± 0.02^C^
Villi	Diet	C	1.90 ± 0.04^A^
		HI5	1.87 ± 0.04^A^
		HI10	1.72 ± 0.04^B^
		HI15	1.66 ± 0.04^B^
	Mucin type	Neutral	1.79 ± 0.04^ab^
		Acidic sialylated	1.84 ± 0.04^a^
		Acidic sulfated	1.74 ± 0.03^b^
	Gut segment	Duodenum	1.23 ± 0.03^C^
		Jejunum	1.95 ± 0.04^B^
		Ileum	2.37 ± 0.04^A^
	Fragment	Base	1.75 ± 0.03
		Midsection	1.81 ± 0.04
		Tip	1.80 ± 0.04

*C* control, *HI5* 5% inclusion level of *Hermetia illucens*, *HI10* 10% inclusion level of *Hermetia illucens*, *HI15* 15% inclusion level of *Hermetia illucens*

^1^Data are represented as mean of counts ± SEM

^2^Means with different superscript letters (a, b or A, B, C) within the same column per predictor (i.e. diet, mucin type, gut segment or fragment) differ significantly (*P* < 0.05 or *P* < 0.01, respectively)

**Fig. 5:**
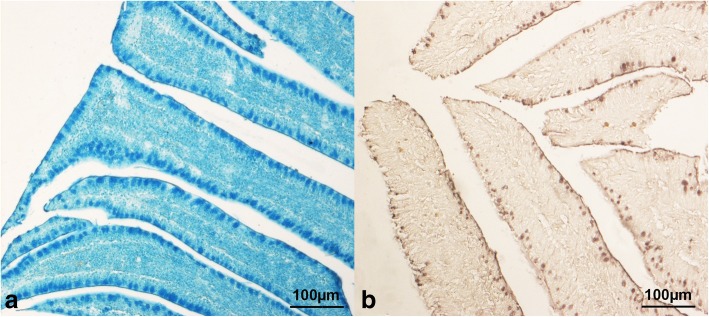
Histological pictures of ileal villi stained with (**a**) Alcian Blue pH 2.5 (HI15 group, 10× magnification) and (**b**) high iron diamine (C group, 10× magnification). Villi show higher acidic sialylated mucin staining intensity than the acidic sulfated

The mucin staining intensity in the intestinal villi of the broiler chickens significantly depended on the dietary treatment (*P* < 0.001) and the gut segment (*P* < 0.001), whereas there was no significant influence of both the mucin type and the villus fragment (*P* > 0.05) on the histochemical scores (Table 1). In particular, the villi of the HI10 and the HI15 animals showed lower mucin staining intensity (*P* < 0.01) than C and HI5. Greater acidic sialylated mucin staining intensity (*P* < 0.05) than the acidic sulfated was also observed (Fig. 5). Furthermore, the ileal villi showed higher mucin staining intensity (*P* < 0.001) than the other gut segments, with a significant increase (*P* < 0.001) being also identified from the duodenum to the jejunum (Table 2).

## Discussion

In the current research, the attention was focused on the cecal microbiota as chicken ceca harbor the highest microbial cell densities and diversity, have the longest residence time of digesta in the gastrointestinal tract, and are an important site for recycling of urea, water regulation, and carbohydrate fermentations contributing to intestinal health and nutrition [26].

Firmicutes, Proteobacteria and Bacteroidetes represented the most abundant bacterial phyla detected in the cecal microbiota of both the C- and the HI-fed broiler chickens of the present study. The predominance of Firmicutes over Bacteroidetes is in agreement with the previous researches [26–29], while the predominance of Proteobacteria over Bacteroidetes clearly contrasts and appears difficult to explain. The Proteobacteria phylum comprises many pathogenic bacteria, such as *Escherichia coli*, *Salmonella* spp., *Vibrio cholera* and *Helicobacter* spp. Furthermore, in human patients high numbers of Proteobacteria members are usually indicative of a bad intestinal health and have a crucial role in the development of some gastrointestinal health conditions such as the chronic dysbiosis [30] and the inflammatory bowel disease [31]. However, an important aspect that must always be taken into consideration is that several host- (i.e., age, sex and breed) and environmental-related factors (i.e., biosecurity level, housing, litter, feed access and climate) may widely influence the chicken intestinal microbiota [32], thus potentially explaining the differences between the current and the previous researches. As a partial confirmation of this aspect, Biasato et al. [17] observed an unexpected predominance of the phylum Bacteroidetes over Firmicutes in female broiler chickens fed both the C- and the TM-based diets.

The cecal microbiota of the birds fed either the C or the HI-based diets in the present study was mainly colonized by Clostridiales order, members of Ruminococcaceae, *Faecalibacterium* and *Oscillospira* genera, and Lachnospiraceae family. These findings reflect the currently available literature, where the main bacterial genera identified in the chicken cecum have been reported to be *Clostridium*, *Ruminococcus*, *Lactobacillus*, *Bacteroides* [28, 33–36] and, to a lesser extent, *Alistipes* and *Faecalibacterium* [28]. The identification of a physiological cecal community also confirms what was observed in a previous research about dietary TM meal inclusion in diets for broilers [17].

Investigating the differences in the 16S rRNA gene sequences between the C- and the HI-fed broiler chickens of the current research, the first aspect to consider is that birds fed the 15% level of HI meal inclusion showed lower Shannon index when compared to the other diets. Differently, higher β-diversity was observed in the broiler chickens fed HI-based diets than C (with a particularly evident distinction between the broilers fed the 5% level of HI meal inclusion and the other diets), as already reported for insect-fed laying hens [37], free-range poultry [16] and broiler chickens [17]. High levels of bacterial diversity have been associated with a maintained stability of the intestinal microbiota after environmental stress factors [38], as well as an effective colonization resistance against the potential pathogenic bacteria [39]. Based on these considerations, insect meal utilization (especially at low inclusion levels) may be advantageous for modulating the complexity of the chicken intestinal microbiota.

Despite no significant differences being observed in regards to phyla composition between the birds fed the C and the HI-based diets in the present study, a specific signature at genus level was, however, detected in their cecal microbiota. In particular, bacteria capable of producing several end products that may affect the intestinal health were identified [40].

In regards to the broiler chickens fed the C diet, Lachnospiraceae family was observed as characteristic OTU of their cecal microbiota. It is well known that Lachnospiraceae, along with Ruminococcaceae, is a typical butyrate-producing family [41]. Butyrate has various positive properties, since it represents an important nutrition source for the enterocytes, stimulates the gut mucin production [42] and improves tight-junction integrity [43]. It is also involved in the cellular differentiation and proliferation within the intestinal mucosa [44] and is capable of reducing the inflammatory response as an anti-inflammatory effector [45].

Secondly, the birds fed the 5% level of HI meal inclusion showed L-*Ruminococcus* (*Ruminococcus* belonging to Lachnospiraceae family), *Faecalibacterium*, *Blautia* and *Clostridium* as characteristic OTUs of their cecal microbiota. Analogously to the already described members of Lachnospiraceae family, *Faecalibacterium* genus encompasses members capable of producing butyric acid [46]. Furthermore, *Blautia* is a new genus belonging to the Ruminococcaceae family that can produce short chain fatty acids (SCFAs) through the glucose metabolism and digest cellulose in food [47]. The SCFAs production is fundamental for the optimal intestinal health, since they represent a remarkable source of energy for enterocytes [48] and have the ability to suppress the enteric pathogens [49]. Last but not least, *Clostridium* is one of the main bacterial genera detected in the chicken cecum [28, 33, 34], being also capable of producing butyrate [50]. Increased abundance of *Clostridium* genus has also been reported in TM-fed broiler chickens [17].

In regards to the broiler chickens fed the 10% level of HI meal inclusion, *Lactobacillus* and *Ruminococcus* were identified as characteristic OTUs of their cecal microbiota. These bacterial genera are frequently identified in the normal chicken microbiota [28, 33–36]. However, the most relevant finding is that *Lactobacillus* positively stimulates the homeostasis of immune cells and the host gut health [51, 52]. The lactate produced by *Lactobacillus* species can also be converted to SCFAs [53–55], whose positive properties have already been described. Interestingly, TM meal utilization has previously been reported to reduce the relative abundance of *Ruminococcus* genus in the chicken cecal microbiota [17], thus suggesting a potential different way of action of the two insect types.

As a final aspect to consider, the birds fed the 15% level of HI meal inclusion showed *Bacteroides*, *Roseburia* and *Helicobacter* as characteristic OTUs of their cecal microbiota. Apart from being one of the most predominant members of chicken microbiota [28, 33–36], *Bacteroides* genus may significantly contribute to the gut health of the animals. Its positive effects are related to its beneficial role for weight gain and growth performance [56] and the inhibition of *Clostridium perfringens* sporulation by its fermentation products [57]. Furthermore, *Roseburia* is a well-known butyrate-producing genus [58], thus further representing another potential beneficial bacterium. However, a potential negative finding may be represented by the remarkable identification of *Helicobacter* genus. Indeed, some specific enterohepatic *Helicobacter* species (i.e., *Helicobacter pullorum*) have been detected in gut and liver of hens with vibrionic-like liver lesions and humans with gastroenteritis [59]. Furthermore, bacteria such as *Helicobacter pylori* possess the enzymatic ability to disrupt the oligomeric structure of mucin and are capable of down-regulating the mucin synthesis [8]. Interestingly, the animals fed the 10% and 15% levels of HI meal inclusion (especially the 15%) showed reduced mucin production in the intestinal villi, thus suggesting a direct interaction between the microbiota and the mucin dynamics. This also confirms what was recently reported in broilers chickens diets containing the 10% inclusion level of TM meal, which displayed decrease in villi mucins and high abundance of *Helicobacter* genus [17].

In the current research, higher mucin staining intensity was observed in the intestinal villi of the broiler chickens fed the 5% level of HI meal inclusion when compared to the 10% and 15%, with the latter also showing lower mucin staining intensity than the C group. Forder et al. [60] pointed out that the microbial flora may influence the mucin production, since some bacteria (i.e., *Helicobacter pylori*) are known to possess a strong mucolytic activity [7] that induces the chicken gut to increase the sialomucin production as defense strategy [60]. As already mentioned before, the birds fed the 15% level of HI meal inclusion displayed *Helicobacter* genus as one of the characteristic OTUs of their cecal microbiota. Therefore, a direct relationship between the reduced intestinal mucin production and the identified bacterial population appears to be reasonable. Mucins also constitute a digestion- and absorption-assisting medium and protect the gut environment against the pathogenic bacteria [5]. Therefore, independently of the intestinal microbiota changes, the utilization of HI meal at low inclusion rates (i.e., 5%) may be preferable to preserve the protective properties of the mucin glycoproteins in order to optimize the digestive process and to prevent the enteric infections, as already suggested by Biasato et al. [17].

Independently of HI meal utilization, the intestinal crypts of the broiler chickens in the present study showed lower acidic sulfated mucin staining intensity than other mucin types. Greater acidic sialylated mucin staining intensity than acidic sulfated was also observed in the intestinal villi. Despite the limited information currently available about the mucin dynamics in crypts and villi, the physiological relevance of the different mucin subtypes has, however, been suggested. In particular, the production of neutral mucins has been recognized as a protective mechanism against the enteric pathogens [61] and as a promoter of the intestinal maturity for facilitating the complex carbohydrate breakdown [60]. Sialic acid groups have also several protective properties [62] and increase in acidic sialylated mucins production has been hypothesized to represent a defense strategy against mucus degradation by bacterial colonization [60]. Finally, a high degree of sulfation within the acidic mucins is also characteristic for immature goblet cells [63]. Therefore, the assessment of mucin types in the current research is indicative of overall mature and healthy guts with a well-developed mucin secretory architecture.

Independently of insect meal inclusion, both the intestinal crypts and the villi of the birds in the present study showed higher mucin staining intensity in the ileum compared with the other gut segments. This is in agreement with the previous findings in chickens, which revealed an increased density of the goblet cells from the duodenum to the ileum [16, 17, 60, 64]. Since the distal ileum has been suggested as a preferred region for bacterial colonization, the above-mentioned mucin dynamics may reflect the need for great protection and subsequent high mucin synthesis [60].

The intestinal crypts of the broiler chickens fed both the C and the HI-based diets in the current research showed greater mucin staining intensity in the base compared with the other crypt fragments. A decreased stain in the tip represents the physiological condition in the intestinal crypts, as previously reported [10, 16, 17, 65]. On the contrary, the intestinal villi showed unaffected mucin staining among the villus fragments. This finding disagrees with what was reported by Tsirtsikos et al. [10, 11], which found greater staining intensity at the villus tip and explained this scenario as a consequence of the key role of mucins in the gut epithelium [10, 11]. However, the goblet cell proliferation may also occur along the entire length of the villus, thus potentially explaining the absence of differences among the villus fragments [65].

As final considerations, the changes in the cecal microbiota and the mucin dynamics observed in the present study may be attributed to both direct and indirect effects of HI meal. The positive increase in SCFAs-producing bacteria predominantly identified in the HI5- and HI10-fed birds could be related to their capability of directly degrading the chitin contained in the insect meal, as already suggested by Borrelli et al. [37]. On the contrary, the proliferation of potential mucolytic pathogens (with the subsequent reduction of villi mucins) observed in the HI15-fed broilers may indirectly be attributed to the increased dietary content of chitin, which can negatively affect the protein digestibility (as already suggested by Dabbou et al. [18]). Indeed, the increase in nondigested protein at ileal level can lead to hindgut protein fermentation, with formation of toxic compounds potentially capable of creating a non-healthy gut environment. Since the CP digestibility of the HI meal used in the current trial was also moderate (0.62) [19], this explanation seems reasonable. Another aspect to recall in relation to the gut mucosal characteristics of the birds in the present study is that the broiler chickens fed the 15% level of HI meal inclusion also showed the worst gut morphology in terms of short villi, deep crypts and reduced villus height to crypt depth ratios. Furthermore, the same birds displayed worse growth performance than the other chickens in terms of higher feed conversion ratios [18]. Since the rapid growth of chickens directly depends on the morphological and the functional characteristics of the digestive tract [66], the relationship between the negative gut microbiota, morphology and mucin composition findings and the deterioration of the growth performance observed in the HI15 birds of the current research seems logical, as already suggested by Biasato et al. [16].

## Conclusions

In conclusion, dietary HI meal inclusion was demonstrated to modulate both the cecal microbiota and the gut mucin composition of the broiler chickens. In particular, insect meal utilization at low inclusion levels (i.e., 5%) positively influenced either the cecal microbiota or the intestinal mucin dynamics in terms of preservation of physiological microbial populations, selection of potentially beneficial bacteria and increase in villi mucins. However, high inclusion levels (in particular the 15%) may have a negative influence in terms of partial reduction of the microbial complexity, reduction of potentially beneficial bacteria, selection of bacteria with mucolytic activity and decrease in villi mucins. In particular, changes in butyrate- and SCFAs-producing bacteria seemed to have a crucial role, but further studies also adopting metatranscriptomic and meta-metabolomic approaches are mandatory to better contextualize these findings. Despite the observed potential negative modulation, the detection of physiological cecal community and intestinal mucin dynamics in all the animals (observed independently of insect meal utilization) represents a positive result in terms of gut health preservation and further stimulates the use of insects in poultry feeding.

## Supplementary information

**Additional file 1.** Ingredients and chemical composition of the experimental diets. Mineral-vitamin premix: vitamin A (retinyl acetate), 12,500 IU; vitamin D_3_ (cholecalciferol), 3000 IU; vitamin E (DL-a-tocopheryl acetate), 60 IU; vitamin K (menadione sodium bisulfite), 1.02 mg; riboflavin, 2.0 mg; pantothenate, 8.0 mg; niacin, 6 mg; piridossin, 4 mg; folic acid, 0.5 mg; biotin, 0.10 mg; tiamin, 1.0 mg; vitamin B_12_, 20 mg; Mn, 120 mg; Zn, 80 mg; Fe, 52 mg; Cu, 15 mg; I, 1.5 mg; Se, 0.4 mg. AME = apparent metabolizable energy; CP = crude protein; EE = ether extract; CF = crude fiber; C = control; HI5 = 5% inclusion level of *Hermetia illucens*; HI10 = 10% inclusion level of *Hermetia illucens*; HI15 = 15% inclusion level of *Hermetia illucens*.

**Additional file 2.** Relative abundance of the main bacterial phyla and genera of cecal microbiota of broiler chickens fed with control (C), 5% (HI5), 10% (HI10) and 15% (HI15) inclusion level of *Hermetia illucens* meal diets.

## Data Availability

The datasets analysed in the present study are available from the corresponding author on reasonable request.

## Abbreviations

AME: Apparent metabolizable energy
AMEn: Apparent metabolizable energy corrected for nitrogen balance
CP: Crude protein
DM: Dry matter
EE: Ether extract
FDR: False discovery rate
HI: *Hermetia illucens*
OTU: Operational taxonomic unit
PCA: Principal component analysis
SCFAs: Short chain fatty acids
SEM: Standard error of the mean
TM: *Tenebrio molitor*
Vh: Villus height
Vh/Cd: Villus height to crypt depth ratio
WG: Weight gain
